# Effects of Precipitant and pH on Coprecipitation of Nanosized Co-Cr-V Alloy Powders

**DOI:** 10.3390/ma10101108

**Published:** 2017-09-21

**Authors:** Xiaoyu Chen, Yongxia Li, Lan Huang, Dan Zou, Enxi Wu, Yanjun Liu, Yuanyan Xie, Rui Yao, Songyi Liao, Guangrong Wang, Feng Zheng

**Affiliations:** 1 Hunan Boyun-Dongfang Powder Metallurgy Co., Ltd., Changsha 410083, China; ChenXY@csu.edu.cn (X.C.); liyongxia1959@hnboyun.com.cn (Y.L.); lhuang@csu.edu.cn (L.H.); zdlyl@189.cn (D.Z.); wuenxi@163.com (E.W.); bydflyj@163.com (Y.L.); mingjian004@126.com (Y.X.); yaorui205@csu.edu.cn (R.Y.); songyiliao@csu.edu.cn (S.L.); g3504251992@163.com (G.W.); 2 School of Materials Science and Engineering, Central South University, Changsha 410083, China; 3 State Key Laboratory of Powder Metallurgy, Central South University, Changsha 410083, China

**Keywords:** Co-Cr-V alloy, morphology coarsening, coprecipitation, precursor, nanosize

## Abstract

Nanosized Co-Cr-V alloy powders were synthesized via coprecipitation method. Effects of precipitants ((NH_4_)_2_C_2_O_4_·H_2_O and Na_2_CO_3_) and pH were investigated by X-ray diffraction (XRD), Zeta potential analyzer, thermogravimetry-differential scanning calorimetry (TG-DSC), inductively coupled plasma-atomic emission spectrometry (ICP-AES) and scanning electron microscopy (SEM). Co-Cr-V alloy powders were consisted of major face-centered cubic Co (fcc Co) and minor hexagonal close-packed Co (hcp Co). Grain sizes of precursors and Co-Cr-V alloy powders were increased with pH value (7–10) within the ranges of 3~39 and 39~66 nm, respectively. Rod-like or granular Co-Cr-V alloy particles were assembled by interconnected nanograins. At pH = 7, Na_2_CO_3_ precipitant was found to be beneficial to maintain the desirable composition of Co-Cr-V powders. It was also found that lower pH favors the maintenance of pre-designed composition, while grain coarsens at higher pH. Effects of variation for precipitant and pH on the morphology and composition of Co-Cr-V alloy powder were discussed in detail and relevant mechanism was further proposed.

## 1. Introduction

Possessing many unique properties such as impressive fatigue and creep strength at high temperature [1,2], good biocompatibility [3] and excellent resistance to oxidation [4], corrosion [2,5], and sulfidation [2,5], cobalt-based alloys are widely used as wear resistant, magnetic, high strength materials and catalysts [6,7,8,9,10]. Effects of alloying elements such as Ni, Cr, Ta, W, Nb, and Mo have been studied by numerous researchers over the last half century. 

Addition of 20~30 at % Cr into cobalt metal can provide superior oxidation and hot corrosion resistance [11]. Co-Cr based alloys have also found utilization as suitable magnetic recording and biomedical applications [12,13]. Similarly, Co-V system is a potential candidate of ultra-high density recording media due to separation between ferromagnetic hcp phase and non-magnetic hcp phase [14]. According to isothermal sections of Co-Cr-Vternary phase diagrams at 25, 800, 1000, 1100, 1150, and 1200 °C [15], adding 0~10 at % of both Cr and V into cobalt metal simultaneously may also lead to phase separation as that observed in Co-V system. These findings suggested that Co-Cr-V ternary alloys should be of great promise as either structural or magnetic recording materials. 

Due to various favorable nanosize-related effects, extensive efforts have been made recently to explore the dependence of material properties on composition and size of cobalt-based alloy nanoparticles mainly in binary systems with different morphologies such as nanowires [16,17], nanotubes [18] and nanorings [2], etc. A variety of synthesis methods, including coprecipitation route [19], hydrothermal/solvethermal [20,21], polyolprocess [22], electrodeposition [18], double composite template approach [23], mechanical alloying [24], chemical vapor deposition [25], and nonaqueous ethylene glycol refluxing [26], have been explored in those works. Coprecipitation route has been proved to be one of the feasible ways to produce highly dispersed, well inter-mixed and uniform cobalt-based alloy nanoparticles [27]. However, despite high industrial and scientific importance for nanosize Co-Cr-V alloy particles stated above, no such work associated with their synthesis, to the best of our knowledge, has been reported up to date.

The objective of the present paper is to study the effects of precipitant and pH on material properties of Co-Cr-V nanoparticles derived from coprecipitation route. Crystal structure, grain size, phase content, morphology and thermal behavior of precursors and final products will be examined and discussed in details.

## 2. Materials and Methods

### 2.1. Materials

All chemicals used here are of analytical grade produced by Xilong Chemical Co. Ltd. (Shantou, China) without further purification. CoCl_2_·6H_2_O, CrCl_3_·6H_2_O and NH_4_VO_3_ were chosen and acted as sources of Co, Cr and V, respectively. Two different precipitants, i.e., (NH_4_)_2_C_2_O_4_·H_2_O and Na_2_CO_3_, were employed to compare their effects on physicochemical properties of Co-Cr-V alloy powders. Aqueous ammonia was added to adjust pH value and PVP was employed as dispersant.

### 2.2. Synthesis

**Figure 1:**
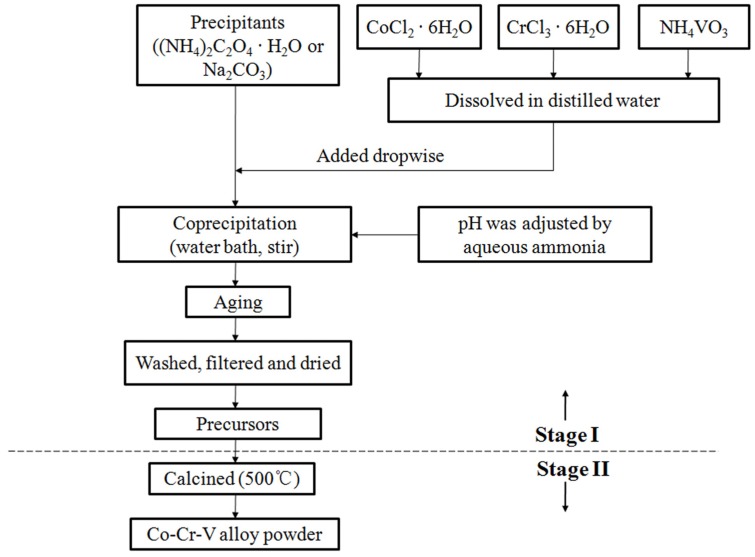
Flow chart of synthesis.

Typical synthesis of Co-Cr-V alloy powders (shown in Figure 1) was performed in two basic stages: (I) preparation of precursors by wet chemical coprecipitation method, and (II) heat treatment of precursors to obtain Co-Cr-V alloy powders.

There are six steps involved in Stage I:

(1) CoCl_2_·6H_2_O, CrCl_3_·6H_2_O and NH_4_VO_3_ were dissolved in distilled water at molar ratio of Co:Cr:V = 8:1:1 to prepare mixed salt solution of 0.5 mol/L with addition of 2 mol % PVP as dispersant.
(2) The above solution was equally divided into six parts for later use.
(3) Each part (of the solution) was dropped (4 mL/min) into aqueous solution of precipitant (1 mol/L (NH_4_)_2_C_2_O_4_·H_2_O or Na_2_CO_3_ solution) at designated pH value via constant pressure funnel. Excessive precipitant by 20 mol % was used to ensure fully precipitation of metallic ions.
(4) Coprecipitation (i.e., Step 3) was thermostated in water bath at 50 °C for 1 h with continuous magnetic stirring. pH value of the solution was monitored (by pH meter) and maintained as constant by adding aqueous ammonia.
(5) Solution with precipitates after coprecipitation was aged in open air for 12 h at room temperature.
(6) As-prepared precipitates were washed with deionized water by filtrating (filtrates were collected for subsequent testing), dried at 80 °C overnight to obtain precursors denoted with PO for oxalates and PC for basic carbonates.

Stage II: Both oxalate and basic carbonate precursors were kept at 500 °C (heating rate of 5 °C/min) for 3 h in hydrogen atmosphere, then cooled down to room temperature to get Co-Cr-V alloy powders, and denoted as AO and AC, accordingly.

All samples including precursors and alloy powders were named according to their synthesis parameters, as presented in Table 1.

### 2.3. Characterization

Phases of precursors and alloy powders were determined by X-ray diffraction (XRD) analysis using an X-ray diffractometer (D/Max 2500, Rigaku, Japan) with CuKα radiation (λ = 0.154060 Å). Grain size was calculated through Scherrer Equation by the strongest peak:

(1) $$d=\frac{K\lambda }{\beta_{1/2}\cos\theta }$$

where d is the mean crystallite diameter, the X-ray wave length, K the Scherrer constant (0.89), 1/2 the full-width at half-maximum (FWHM) of the main diffraction peak of crystalline phase and the diffraction angle. Relative phase content of alloy powders and their lattice parameters were obtained by Rietveld method using Jade^®^ 6.0 program. Zeta potentials at different pH were measured by Zeta potential analyzer (Zetasizer Nano ZS, Malvern, UK). Thermogravimetry (TG) and differential scanning calorimetry (DSC) curves of precursors were measured and recorded by DSC200F3 Maia (NETZSCH, Bavaria, Germany) between 35 and 700 °C at heating rate of 10 °C/min in argon atmosphere. Residual concentrations of metallic ions in filtrates were determined by inductively coupled plasma-atomic emission spectrometry (ICP-AES, PS-6, Baird, Milwaukee, WI, USA). Morphology of all powder samples were examined by scanning electron microscopy (SEM, Quanta-200, FEI, Hillsboro, OR, USA).

## 3. Results and Discussion

### 3.1. XRD Analysis

**Figure 2:**
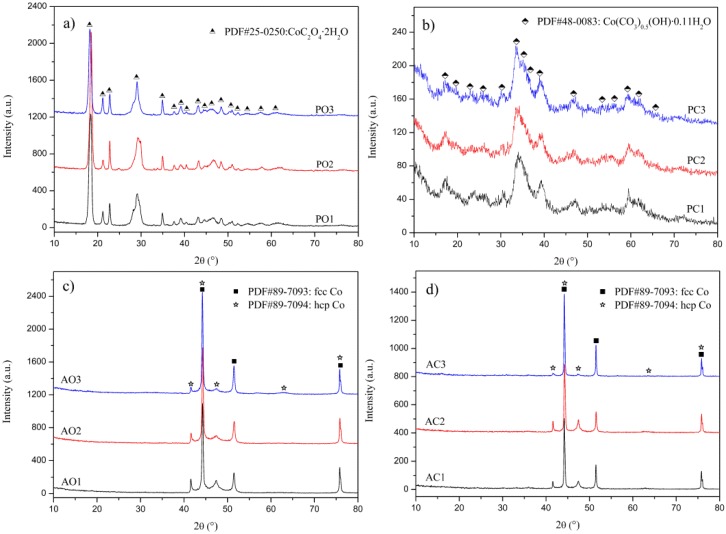
XRD patterns of :(**a**,**b**) precursors; and (**c**,**d**) Co-Cr-V alloy powders.

XRD patterns of precursors and their thermally decomposed products (alloy powders) are presented in Figure 2a–d. All diffraction peaks for samples PO1–PO3 were corresponding to cobalt oxalate (CoC_2_O_4_·2H_2_O, JCPDS No. 25-0250), and those for samples PC1–PC3 were indexed as basic cobalt carbonate (Co(CO_3_)_0.5_(OH)·0.11H_2_O, JCPDS No. 48-0083). No secondary phase was detectable here. All products heated at 500 °C with flowing hydrogen from either oxalate (AO1–AO3) or basic carbonate precursors (AC1–AC3) yielded a mixture of two types of cobalt phases, i.e., fcc Co (JCPDS No. 89-7093) and hcp Co (JCPDS No. 89-7094). No peak can be assigned to either Cr- or V-containing phase. 

Lattice parameters of alloy powders were obtained by Rietveld analysis and listed in Table 2. We noted lattice distortions for both fcc Co and hcp Co phases in all samples by comparing their lattice parameters with those of powder diffraction files (PDFs) inside Jade^®^ 6.0. Considering atomic radius of Co (1.26 Å), Cr (1.27 Å) and V (1.35 Å), substitution of Co by Cr and especially V will necessarily lead to expansion of crystal lattice. Our data listed in Table 2 showed apparent expansion along three dimensions for fcc Co phase and c-axis for hcp Co phase, indicating that Cr and/or V should have dissolved into Co matrix. Moreover, composition of alloy powders confirmed the existence of both Cr and V together with Co (detailed discussion will be provided later in Section 3.3), which lead us to believe and conclude that Co have been partially substituted by Cr and V to form solid solution, which is in agreement with Co-Cr-V ternary phase diagrams [15]. These also implied that, during aqueous reaction stage, both Cr and V ions have simultaneously precipitated with those of Co to form single-phased precursor.

Considering the fact that there was no oxidation-reduction reaction occurred during coprecipitation, it is then believed that Cr and V ions should maintain their original valence states of 3^+^ and 5^+^ in precipitates, respectively. To maintain charge neutrality, some vacancies must form and exist in precipitates. In other words, precipitates could be expressed as (Co*_x_*_1_Cr*_x_*_2_V*_x_*_3_□*_x_*_4_)C_2_O_4_·2H_2_O and (Co*_y_*_1_Cr*_y_*_2_V*_y_*_3_□*_y_*_4_)(CO_3_)_0.5_(OH)·0.11H_2_O for oxalate and basic carbonate precursors, accordingly, with □representing for vacancy. Detailed calculation of the two sets of variables, *x*1–*x*4 and *y*1–*y*4, will be discussed in Section 3.3.

**Figure 3:**
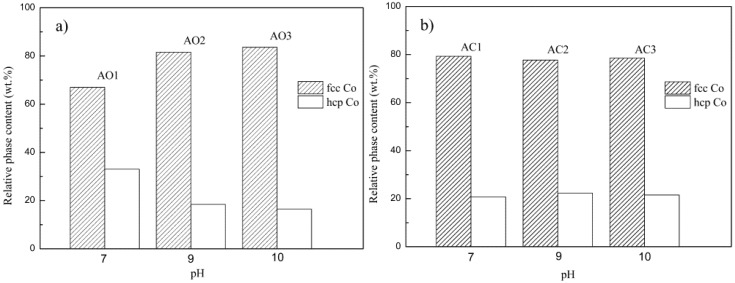
Relative phase content of fcc Co and hcp Co in samples of: (**a**) AO1–AO3; and (**b**) AC1–AC3.

Relative phase contents of both fcc Co and hcp Co in alloy powders were calculated from XRD patterns, as given in Figure 3a,b. It turned out that fcc Co phase is dominant for all samples, indicating that fcc Co phase is stable at room temperature here. This is, interestingly, different from knowledge for conventional coarse-grain Co metal, whereas the stable phase is hcp Co [28]. The reason for stabilization of fcc Co at room temperature will be discussed later.

Average grain sizes of all samples calculated from Scherrer equation using full-width at half-maximum of the strongest diffraction peak (202) for oxalate, (221) for basic carbonate precursors, (111) for fcc Co, and (101) for hcp Co are tabulated in Table 3. Results show that average grain sizes of oxalate and basic carbonate precursors increased with pH value and ranged 13~39 and 3~6 nm, respectively.

There is no universal mechanism in the literature for the effects of pH variation on grain growth for salt solution. However, it has been well documented that grain growth and particles agglomeration are controlled effectively by change of pH value of precipitating medium [29,30,31]. Nucleation and grain growth in aqueous solution are affected by the presence of different types of ions, their concentrations and interactions as well as intrinsic properties of precipitates. In our case, samples were prepared in solutions with different pH value where concentrations of H^+^ and OH^−^ varied from each other. Solution at higher pH yields higher concentration of OH^−^, which will drive ionic equilibrium of C_2_O_4_^2^^−^ or CO_3_^2^^−^ (Equations (2)–(5)) to shift towards left, resulting in higher content of C_2_O_4_^2^^−^ or CO_3_^2^^−^.

C_2_O_4_^2^^−^ + H_2_O ⇋ HC_2_O_4_^−^ + OH^−^ (2)

HC_2_O_4_^−^ + H_2_O ⇋ H_2_C_2_O_4_ + OH^−^ (3)

or

CO_3_^2^^−^ + H_2_O ⇋ HCO_3_^−^ + OH^−^ (4)

HCO_3_^−^ + H_2_O ⇋ H_2_CO_3_ + OH^−^ (5)

**Figure 4:**
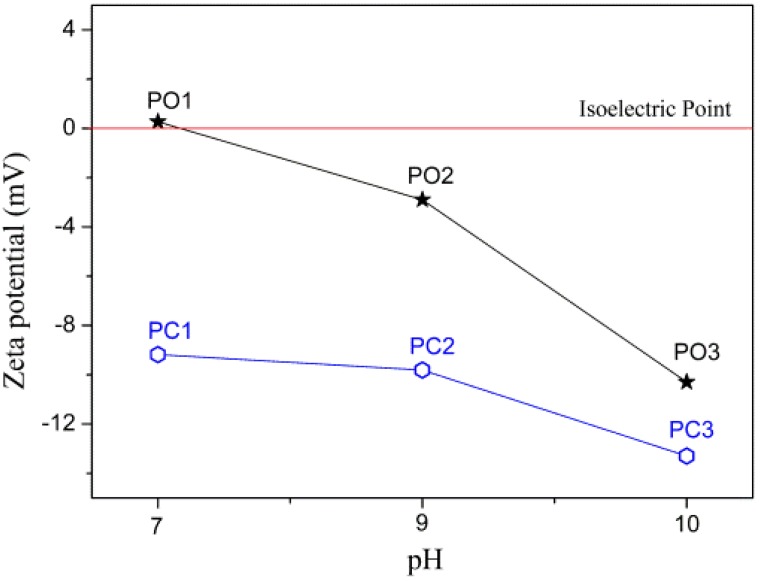
Zeta potential of filtrates as function of pH value.

On the one hand, once there are larger amount of C_2_O_4_^2^^−^ or CO_3_^2^^−^ in the solution system at higher pH value, the frequency of collision among ions of reactants will be increased and thereby boost grain growth, leading to increase of grain size with pH value under low supersaturation (metal salts were added dropwise). On the other hand, we supposed that surfaces of precipitates would attain stronger negative charges at higher pH due to absorption of C_2_O_4_^2^^−^ or CO_3_^2^^−^, resulting in larger potential difference between surfaces of precipitates and diffuse layers in solution. To quantitatively analyze potential difference of particles during reaction to examine this hypothesis, Zeta potentials (characterization of potential difference between surface of solid particles and diffuse layers [32]) were measured, as given in Figure 4. In Figure 4, we can see that absolute values of Zeta potential (0.27~10.9 and 9.18~13.3 mV for oxalate and basic carbonate precursors, respectively) did increase with pH, supporting our hypothesis. With larger potential difference between surfaces of precipitates and diffuse layers in solution, diffusion fluxes of metallic ions (Co, Cr and V ions here) and thus grain growth of precipitates would be increased greatly. These allow us to believe that potential difference between particle surface and diffuse layers should also be responsible for grain coarsening.

All alloy powder samples are in nanosize scale (≤ 100 nm) with average grain size ranging from 40 to 60 nm. The tendency of grain size varying with pH is generally consistent with that of precursors. Usually, coarse Co will undergo phase transition of fcc → hcp (around 420 °C) at cooling from high temperature. However, it is suggested that fcc Co could be stabilized and retained to room temperature instead of going through transition to hcp structure if its grain size is smaller than critical size (usually nanometer scale), [33,34]. The exact value of that critical size may depend on experimental conditions, e.g. 20 nm [35] or 35 nm [36]. Considering our case, where most alloy powders are in the lattice structure of fcc Co phase rather than hcp one at room temperature, conclusions could be made such that: (1) the exact critical sizes to retain fcc Co structure here are larger than those obtained in [35,36]; and (2) for each of our samples, most grains are smaller than the critical size, leading to the absence of allotropic transition of fcc Co→hcp Co during cooling to room temperature.

### 3.2. Thermal Behavior of Precursors

**Figure 5:**
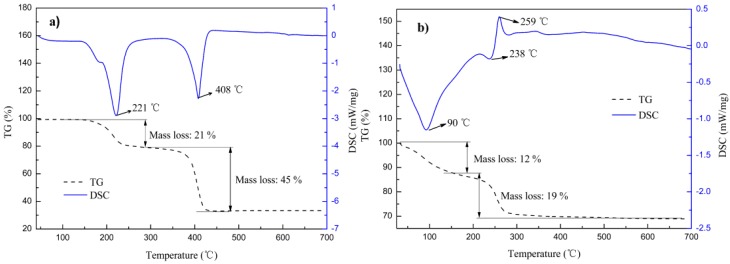
TG and DSC curves for samples of: (**a**) PO1; and (**b**) PC1.

Typical TG and DSC curves of precursors PO1 and PC1 in argon atmosphere are presented in Figure 5a,b, respectively.

For oxalate precursor PO1, two main endothermic peaks took place around 221 and 408 °C accompanied by mass losses of roughly 21% and 45% within 150~300 °C and 300~450 °C, respectively. The first mass loss of 21% is close to that (of 20%) caused by loss of crystal water, while the second loss of 45% agrees approximately with expected value of 48% calculated from decomposition of pure CoC_2_O_4_ to Co. According to XRD analysis, PO1 should be a complex compound (Co*_x_*_1_Cr*_x_*_2_V*_x_*_3_□*_x_*_4_)C_2_O_4_·2H_2_O rather than CoC_2_O_4_·2H_2_O). The mass variation resulted from substitution of Co by Cr and V could be subtle because of: (1) similar atomic mass of Cr (52.00 amu), V (50.94 amu) and Co (58.93 amu); and (2) relatively low content of Cr and V. Hence, the second mass change can be attributed to complete decomposition from (Co*_x_*_1_Cr*_x_*_2_V*_x_*_3_□*_x_*_4_)C_2_O_4_·2H_2_O to Co-based alloy accompany by release of CO_2_. Corresponding phase transition referred to these two mass losses could be described as the following:

CoC_2_O_4_·2H_2_O → CoC_2_O_4_ + 2 H_2_O↑ (150~300 °C) (6)

CoC_2_O_4_ → Co + 2 CO_2_↑ (300~450 °C) (7)

For basic carbonate precursor PC1, two mass losses occurred around 80~150 °C and 150~500 °C, respectively. The first mass loss of 12% is ascribed to dehydration of surface-absorbed water and some structural water with one observable endothermic peak. For Co(CO_3_)_0.5_(OH), it is chemically identical to 1/2[CoCO_3_·Co(OH)_2_], both CoCO_3_ and Co(OH)_2_ can be thermally decomposed into CoO with the former occurred around 400 °C and the latter at 1000 °C in air. Hence, we believed that the second mass loss occurred around 150~500 °C in argon due to removal of CO_2_ by decarbonation from CoCO_3_ to CoO, accompanying mass loss of 19%, which is close to theoretical value of 18% from 1/2[CoCO_3_·Co(OH)_2_] changing to 1/2[CoO·Co(OH)_2_]. Note, the second mass loss was supposed to be an endothermic process. In addition to one endothermic peak occurred at 238 °C here, however, one exothermic peak appeared at 259 °C. We have then inferred that some intermediate product(s) (multiple oxides, for example) might be formed during phase transition, subjecting to oxidation-reduction reaction through self-catalysis with thermal emission. Phase transition of basic carbonate precursor at heat treatment in argon was therefore given as:

Co(CO_3_)_0.5_(OH)·*x*H_2_O → Co(CO_3_)_0.5_(OH) + *x*H_2_O↑ (80~150 °C) (8)

(note: x contains surface-absorbed moisture and some structural water)

Co(CO_3_)_0.5_(OH) → 1/2CoO + 1/2Co(OH)_2_ + 1/2CO_2_↑ (150~500 °C) (9)

In summary, mass losses corresponding to different reaction processes as well as phase evolutions of precursors (PO1 and PC1) have been examined and presented in Table 4. 

According to aforementioned thermal and XRD analysis, we believe that basic carbonate precursor is thermally more stable than that of oxalate one, and using of flowing hydrogen is necessary to form pure Co-Cr-V alloy powders.

### 3.3. Composition Analysis of Precursors and Co-Cr-V Alloy

Maintaining preset molar ratio of Co, Cr and V (8:1:1) is of vital importance for our final products, i.e., Co-Cr-V alloy powders. Their compositions are closely related to pH value during solution reactions. For our solution system consisting of Me-NH_4_^+^-NH_3_-C_2_O_4_^2−^ (or CO_3_^2−^)-H_2_O, diversified coordination reactions would take place besides coprecipitation to consume partial metallic ions to form soluble metal-ammonia complex Me(NH_3_)_n_^x+^, where n and x represents coordination number and valence of metal, respectively. As a result, not all metallic ions went into precipitates and thereby the actual molar ratio of Co, Cr and V might deviate from preset value of 8:1:1. The level of this deviation depends on solubility of precipitates and stability of soluble complexes which may vary with pH value during reaction [37].

**Figure 6:**
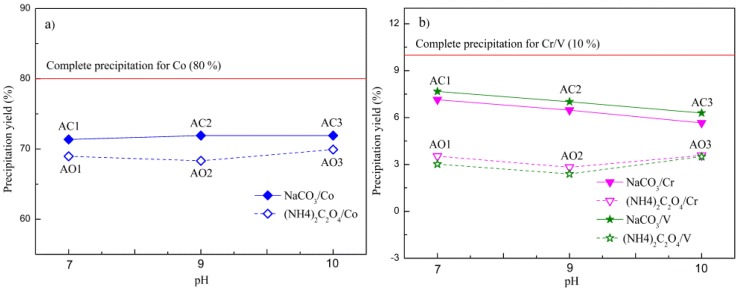
Yields of: (**a**) Co; and (**b**) Cr and V as functions of precipitant and pH.

Careful ICP-AES measurement and calculation on residual concentrations of metallic ions in filtrates from coprecipitation process have revealed the yield of each alloying element as well as chemical composition of precursors and Co-Cr-V alloy powders, as shown in Figure 6a,b (with target yield of Co set to be 80% and that of both Cr and V to be 10%) and listed in Table 5.

According to Co-Cr-V alloy composition (see Table 5) and principle of charge neutrality, the two sets of variables, *x*1–*x*4 and *y*1–*y*4, mentioned in Section 3.1 have been calculated with detailed chemical formulas for oxalate and basic carbonate precursors presented in Table 5.

As illustrated in Figure 6a,b, for both types of precursors, the yield of Co remained almost constant with pH and closed to target yield of 80% at pH = 7~10. While those of Cr and V appeared to be much lower than target ones and sensitive to pH, especially for oxalate precursors with yields of both Cr and V to be about 3% (target yield of 10%). With respect to basic carbonate precursors, the yields of both Cr and V were decreased with pH and in the range of 6~8%. Chemical composition of resulting alloy powders prepared at pH = 7 (AC1) was Co_0.83_Cr_0.08_V_0.09_ with molar ratio of Co, Cr and V to be 8:0.80:0.86, close to predesigned value (8:1:1) than samples obtained at higher pH values. In another word, higher pH values would favor coordination reactions rather than coprecipitation in system containing Me-NH_4_^+^-NH_3_-CO_3_^2^^−^-H_2_O. Hence, more metallic ions would stay in the solution as part of soluble complexes Me(NH_3_)_n_^x+^, preventing precipitation of Cr and V and causing larger deviation of molar ratio (Co:Cr:V) from preset value. Interestingly, different trend was observed for oxalate precursors such that their yields of Cr and V decreased first and then increased with pH. This is believed to be closely related to the variation of stability of those metal-ammonia complexes, which could only be stable at certain pH range and will dissociate to free metallic ions again under high alkaline condition, resuming precipitation, as was the case for AO3 prepared at pH of 10. Generally speaking, Na_2_CO_3_ is a more effective precipitant due to its causing much higher yields of Co, Cr and V than that of (NH_4_)_2_C_2_O_4_·H_2_O. Furthermore, pH = 7 is beneficial to maintain preset composition of Co-Cr-V powders.

### 3.4. Morphology Analysis

**Figure 7:**
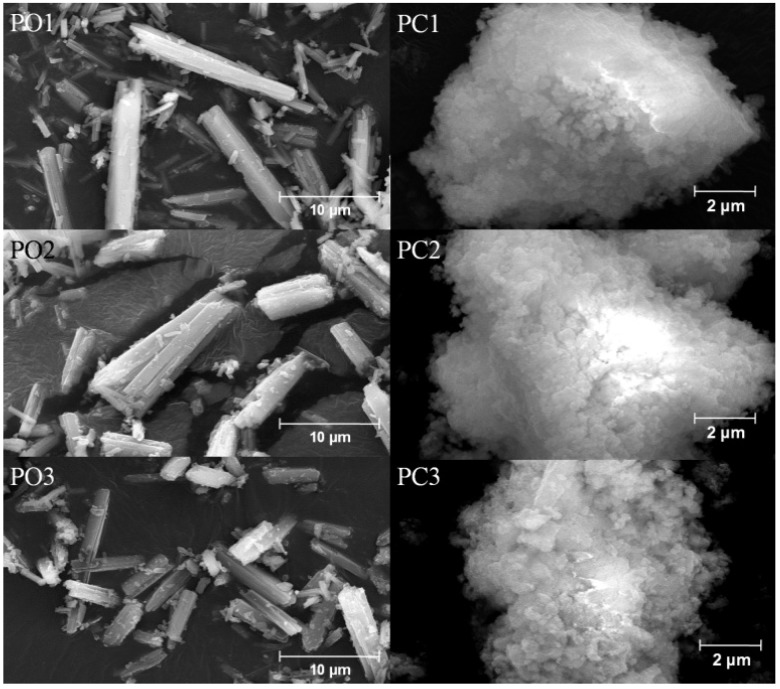
Morphologies of precursors.

**Figure 8:**
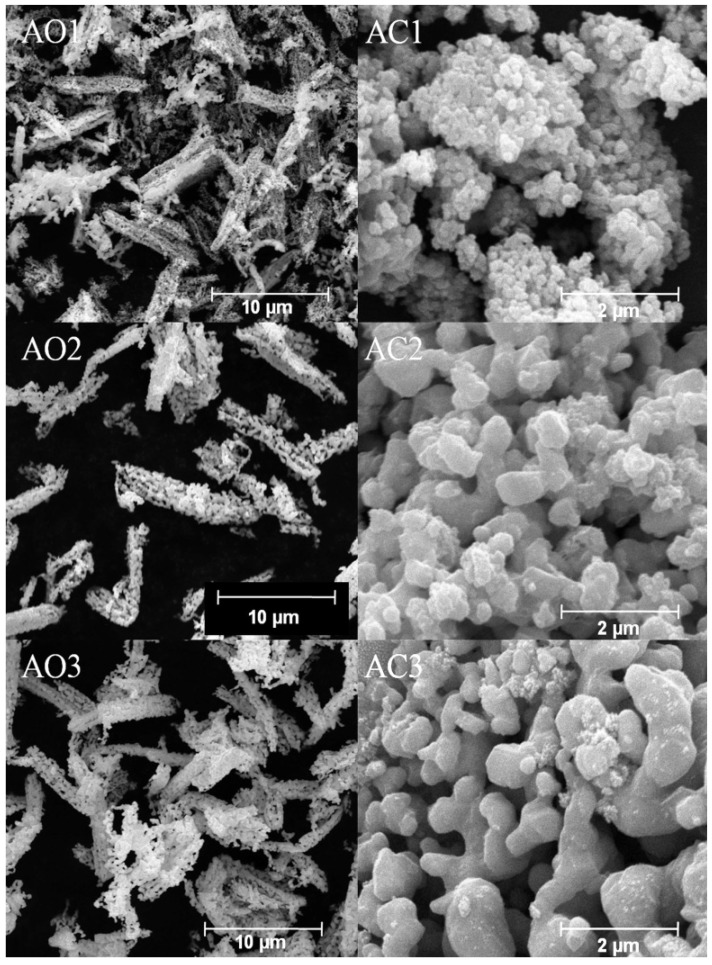
Morphologies of Co-Cr-V alloy powders.

Morphologies of precursors and alloy powders are shown in Figure 7 and Figure 8, respectively. Oxalate precursors (PO1–PO3) are composed of well dispersive rod-like particles with length of 2~10 μm, while basic carbonate precursors (PC1–PC3) appeared as granular agglomerates from fine grains (see Figure 7). The difference in morphologies of those two types of precursors is closely related to different crystal structure of oxalate and basic carbonate molecules [38]. For oxalate precursors, central metal atom was bonded by two C_2_O_4_^2^^−^ ions to form a planar molecule. Perpendicular to the molecular plane there are two coordinated H_2_O molecules by which crystal grain can grow in an elongate way along axial direction, leading to rod-like shapes [39]. For basic carbonate precursors, due to almost equal growing speed along different directions, they then grew into granular shape. Superstructure of Co-Cr-V alloy powders was inherited from their precursors, which can be observable from Figure 8. These alloy powders are in fact formed by a large amount of rather uniform nanoparticles interconnected with each other along original direction of precursors. Formation of new crystallite phase(s) had occurred in situ as such that their relative positions of particles being kept unchanging as those from corresponding precursors. In addition, as indicated in TG-DSC results (Figure 5), there were gaseous H_2_O and CO_2_ released from precursors during heat treatment, which led to disruption of tightly packed rod-like and granular morphologies and resulted in the formation of architectures with nano- and/or submicro-array of particles.

It can be inferred that our Co-Cr-V alloy powders with rod-like morphologies have larger magneto-crystalline anisotropy and therefore larger coercivity compared to granular morphologies. In addition, it is known that coercivities of nanosized magnetic powders are proportional to the sixth power of their diameters [40]. In other words, with adjustable grain size (40~60 nm, see Section 3.1) and morphologies, the magnetic properties, especially coercivity, of nanosized Co-Cr-V alloy powders maybe tunable to satisfy different applications such as ultra-high density magnetic recording media and biomedical materials used in thermotherapy.

## 4. Conclusions

In conclusion, oxalate and basic carbonate precursors and Co-Cr-V alloy powders have been synthesized via coprecipitation method using (NH_4_)_2_C_2_O_4_·H_2_O and Na_2_CO_3_ as precipitants. Grain sizes of precursors and alloy powders were increased with pH and within nanosize ranges, due to equilibrium shift of C_2_O_4_^2^^−^ or CO_3_^2^^−^ inside their solutions, resulting in higher frequency of collision among ions and larger potential difference between particles surfaces and diffuse layers. As-prepared Co-Cr-V alloy powders consisted of major fcc Co and minor hcp Co phases. The presence of fcc Co phase at room temperature could be ascribed to its containing nanosized grains less than 60 nm. Comparative analysis revealed that Na_2_CO_3_ is a more effective precipitant capable of generating higher yields of Co, Cr and V. Increase in pH value from 7 to 10 may favor coordination reactions rather than coprecipitation in solution, causing larger deviation of chemical composition of Co-Cr-V alloy powders from preset value. Rod-like particles and granular agglomerates were obtained for oxalate and basic carbonate precursors, respectively. Morphologies of Co-Cr-V alloy powders inherited from precursors were formed by loosely packed nano- and/or submicro-particles. With adjustable grain size and morphologies, these nanosized Co-Cr-V alloy powders could be used as promising catalytic, magnetic recording or structural materials.

## Figures and Tables

**Table 1:** Synthesis conditions and names of samples.

Samples		Parameters of Synthesis	
Precursor	Alloy Powder	Precipitant	pH Value
PO1	AO1	(NH_4_)_2_C_2_O_4_.H_2_O	7
PO2	AO2		9
PO3	AO3		10
PC1	AC1	Na_2_CO_3_	7
PC2	AC2		9
PC3	AC3		10

**Table 2:** Lattice parameters of alloy powders.

Samples	Lattice Parameters (Å)		
	fcc	hcp	
	A = b = c	a = b	c
AO1	3.5493 ± 0.0010	2.5066 ± 0.0008	4.0853 ± 0.0007
AO2	3.5482 ± 0.0003	2.5068 ± 0.0009	4.0886 ± 0.0009
AO3	3.5463 ± 0.0009	2.5071 ± 0.0012	4.0924 ± 0.0017
AC1	3.5448 ± 0.0007	2.5076 ± 0.0012	4.0989 ± 0.0029
AC2	3.5455 ± 0.0001	2.5063 ± 0.0015	4.0913 ± 0.0036
AC3	3.5461 ± 0.0006	2.5071 ± 0.0010	4.0929 ± 0.0023
JCPDS No.89-7093 (fcc Co)	3.5442	-	-
JCPDS No.89-7094 (hcp Co)	-	2.5074	4.0699

**Table 3:** Grain sizes of precursors and alloy powders.

Precursors	Grain Size (nm)	Alloy Powders	Grain Size (nm)	
			fcc Co	hcp Co
PO1	13	AO1	40	44
PO2	15	AO2	41	46
PO3	39	AO3	48	65
PC1	3	AC1	39	46
PC2	4	AC2	59	66
PC3	6	AC3	43	58

**Table 4:** Thermal decomposition analysis for precursors.

Sample	Temperature (°C)	Massloss (wt %)		Phase Evolution	Thermal Effect
		Exp.	Theor.		
PO1	150~300	21	20	CoC_2_O_4_·2H_2_O → CoC_2_O_4_	Endoth./221 °C
	300~450	45	48	CoC_2_O_4_ → Co	Endoth./408 °C
PC1	80~150	12	/	Co(CO_3_)_0.5_(OH)·*x*H_2_O → Co(CO_3_)_0.5_(OH)	Endoth./90 °C
	150~500	19	18	Co(CO_3_)_0.5_(OH) → CoO + Co(OH)_2_	Endoth./238 °C partial Exoth./259 °C

**Table 5:** Chemical compositions of precursors and Co-Cr-V alloy powders.

Precursor Samples	Calculated Chemical Formula of Precursors	Alloy Samples	Composition of Alloy Powders	Molar Ratio of Co:Cr:V
PO1	(Co_0.84_Cr_0.04_V_0.04_□_0.08_)C_2_O_4_·2H_2_O	AO1	Co_0.91_Cr_0.05_V_0.04_	8:0.41:0.35
PO2	(Co_0.87_Cr_0.04_V_0.03_□_0.06_)C_2_O_4_·2H_2_O	AO2	Co_0.93_Cr_0.04_V_0.03_	8:0.33:0.28
PO3	(Co_0.84_Cr_0.04_V_0.04_□_0.08_)C_2_O_4_·2H_2_O	AO3	Co_0.91_Cr_0.05_V_0.04_	8:0.41:0.40
PC1	(Co_0.71_Cr_0.07_V_0.07_□_0.15_)(CO_3_)_0.5_(OH)·0.11H_2_O	AC1	Co_0.83_Cr_0.08_V_0.09_	8:0.80:0.86
PC2	(Co_0.73_Cr_0.06_V_0.07_□_0.14_)(CO_3_)_0.5_(OH)·0.11H_2_O	AC2	Co_0.84_Cr_0.08_V_0.08_	8:0.72:0.78
PC3	(Co_0.75_Cr_0.06_V_0.06_□_0.13_)(CO_3_)_0.5_(OH)·0.11H_2_O	AC3	Co_0.86_Cr_0.07_V_0.07_	8:0.63:0.70
